# Cholangiocarcinoma associated with limbic encephalitis and early cerebral abnormalities detected by 2-deoxy-2-[fluorine-18]fluoro-d-glucose integrated with computed tomography-positron emission tomography: a case report

**DOI:** 10.1186/s13256-016-0989-1

**Published:** 2016-07-20

**Authors:** Sergio L. Schmidt, Juliana J. Schmidt, Julio C. Tolentino, Carlos G. Ferreira, Sergio A. de Almeida, Regina P. Alvarenga, Eunice N. Simoes, Guilherme J. Schmidt, Nathalie H. S. Canedo, Leila Chimelli

**Affiliations:** 1 State University of Rio de Janeiro, Boulevard 28 de Setembro, 77 - Vila Isabel, Rio de Janeiro, CEP 20551-030 Brazil; 2 Federal University of the State of Rio de Janeiro, Rua Mariz e Barros, 775 - Tijuca, Rio de Janeiro, CEP 20270-901 Brazil; 3 Federal University of Juiz de Fora, Juiz de Fora, Brazil; 4 National Network for Cancer Research, Brazilian Ministry of Health, Brasilia, Brazil; 5 D’Or Institute for Research and Education, Rua Diniz Cordeiro, 30 - Botafogo, Rio de Janeiro, CEP 22281-100 Brazil; 6 Nuclear Medicine, Samaritan Hospital, Rua Bambina, 98 - Botafogo, Rio de Janeiro, CEP 22251-050 Brazil; 7 Anatomic Pathology Service, Federal University of Rio de Janeiro, Rua Prof. Rodolpho Paulo Rocco, 255 Subsolo sala SS F 21 - Ilha do Governador, Rio de Janeiro, CEP 21939-900 Brazil

**Keywords:** Cholangiocarcinoma, Limbic encephalitis, Voltage-gated potassium channel complex antibodies, Cerebral PET/CT, Whole-body PET/CT

## Abstract

**Background:**

Limbic encephalitis was originally described as a rare clinical neuropathological entity involving seizures and neuropsychological disturbances. In this report, we describe cerebral patterns visualized by positron emission tomography in a patient with limbic encephalitis and cholangiocarcinoma. To our knowledge, there is no other description in the literature of cerebral positron emission tomography findings in the setting of limbic encephalitis and subsequent diagnosis of cholangiocarcinoma.

**Case presentation:**

We describe a case of a 77-year-old Caucasian man who exhibited persistent cognitive changes 2 years before his death. A cerebral scan obtained at that time by 2-deoxy-2-[fluorine-18]fluoro**-** d **-**glucose integrated with computed tomography-positron emission tomography showed low radiotracer uptake in the frontal and temporal lobes. Cerebrospinal fluid analysis indicated the presence of voltage-gated potassium channel antibodies. Three months before the patient’s death, a lymph node biopsy indicated a cholangiocarcinoma, and a new cerebral scan obtained by 2-deoxy-2-[fluorine-18]fluoro-d-glucose integrated with computed tomography-positron emission tomography showed an increment in the severity of metabolic deficit in the frontal and parietal lobes, as well as hypometabolism involving the temporal lobes. Two months before the patient’s death, cerebral metastases were detected on a contrast-enhanced computed tomographic scan. Postmortem examination revealed a cholangiocarcinoma with multiple metastases including the lungs and lymph nodes. The patient’s brain weighed 1300 g, and mild cortical atrophy, *ex vacuo* dilation of the ventricles, and mild focal thickening of the cerebellar leptomeninges, which were infiltrated by neoplastic epithelial cells, were observed.

**Conclusions:**

These findings support the need for continued vigilance in malignancy surveillance in patients with limbic encephalitis and early cerebral positron emission tomographic scan abnormalities. The difficulty in early diagnosis of small tumors, such as a cholangiocarcinoma, is discussed in the context of the clinical utility of early cerebral hypometabolism detected by 2-deoxy-2-[fluorine-18]fluoro-d-glucose integrated with computed tomography-positron emission tomography in patients with rapidly progressive dementia.

## Background

Most patients with limbic encephalitis (LE) associated with voltage-gated potassium channel complex antibodies (VGKC-Ab) do not have a tumor. The few cases reported in the literature were associated with thymoma [1], small cell lung cancer [2], or myeloid leukemia [3]. Cholangiocarcinoma is a rare tumor arising from either intrahepatic or extrahepatic bile ducts [4]. The presence of brain metastasis due to cholangiocarcinoma is a rare event [4]. To our knowledge, there is no previous report in the literature describing LE and paraneoplastic association with a cholangiocarcinoma.

## Case presentation

**Fig. 1:**
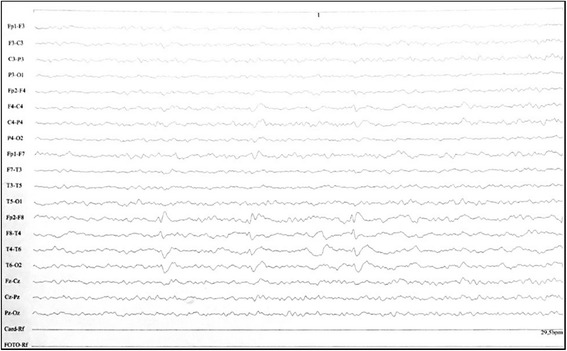
Abnormal electroencephalogram obtained 2 years before the death of the patient. The electroencephalogram shows epileptic discharges from the right temporal lobe

Our patient was a 77-year-old Caucasian man who, 3 years and 11 months before his death, exhibited subacute polyneuropathy that included large fibers with autonomic changes. The clinical presentation of the patient’s polyneuropathy did not allow us to differentiate a paraneoplastic pathology from an atypical presentation of diabetic neuropathy. A first episode of focal epileptic crises occurred 13 months after the polyneuropathy event. Two years before the patient’s death, an electroencephalogram showed epileptic discharges in the right temporal cortex (Fig. 1). After that, his autonomic seizures became more frequent, and ictal pilomotor erection was a common observation.

Two years before the patient’s death, a diagnosis of probable dementia was made. The presence of rapidly progressive dementia, delusions, and autonomic seizures with pilomotor erection suggested a preliminary diagnosis of limbic encephalopathy. At that time, cerebrospinal fluid (CSF) analysis including the following was performed: herpes simplex virus (repeated three times with negative results), varicella zoster virus, cytomegalovirus, Epstein-Barr virus, HIV, human T-cell lymphotropic virus 1/2, JC virus, *Borrelia burgdorferi* (Lyme disease), *Tropheryma whippleii* (detected but not confirmed in a second sample), *Treponema pallidum*, and *Cryptococcus neoformans*. All results of these tests were negative. No growth of any bacteria or fungi was detected. The patient’s protein electrophoresis result was also normal. His 14-3-3 protein test result was negative. The patient had a slightly reduced amyloid-β_42_ level (543 pg/ml). However, his tau protein level was normal (210 pg/ml). His CSF was also analyzed using an established radioimmunoassay in a reference laboratory. Intracellular antibodies (Hu, Ri, Yo, and Ma2) and surface antibodies (VGKCs KV1.1, KV1.2, and KV1.6) were assessed. Negative results were obtained for the intracellular antibodies. According to the relative values provided by the reference laboratory, the results of the VGKC-Ab test were positive.

**Fig. 2:**
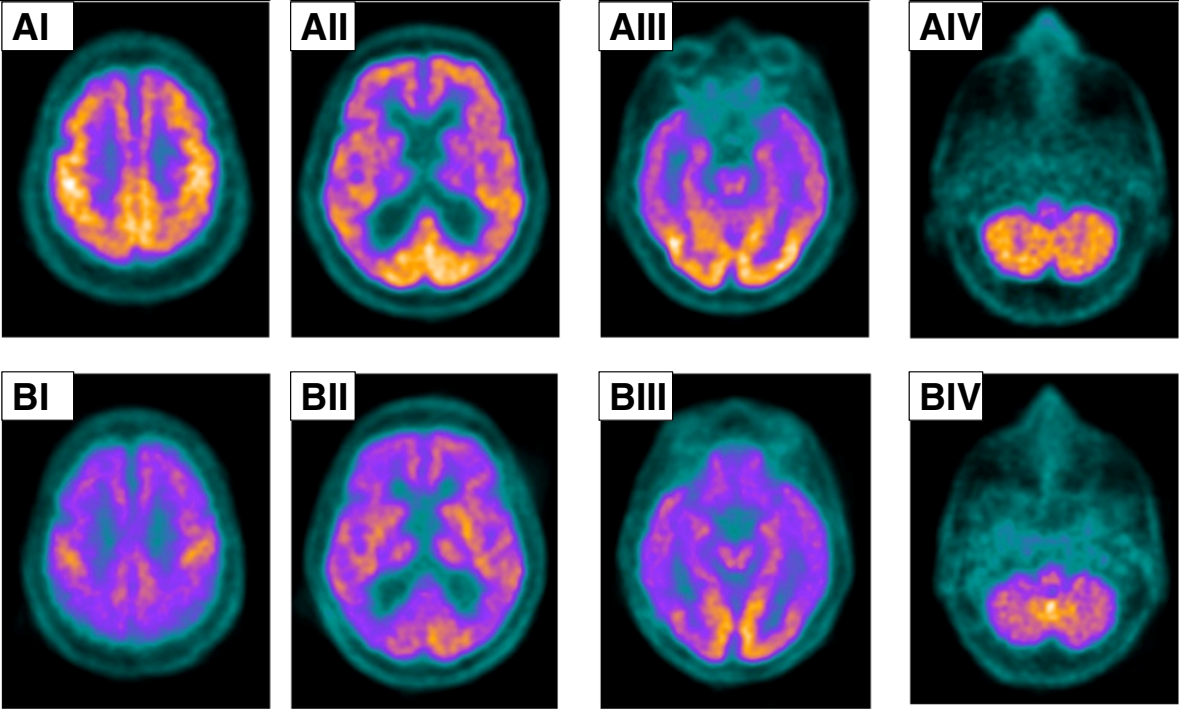
Cerebral scans (axial slices, I–IV) obtained by 2-deoxy-2-[fluorine-18]fluoro-D-glucose integrated with computed tomography-positron emission tomography. Cerebral 2-deoxy-2-[fluorine-18]fluoro-D-glucose integrated with computed tomography-positron emission tomography was performed 23 months before the patient’s death. a Four representative axial slices (AI–AIV) show decreased fluorodeoxyglucose uptake in the frontal and temporal lobes and normal uptake in the posterior cingulum and occipital cortex. Note that the hypometabolism in the affected regions is greater in the right hemisphere. b Cerebral 2-deoxy-2-[fluorine-18]fluoro-D-glucose integrated with computed tomography-positron emission tomography performed 3 months before the patient’s death shows the general reduction in radiotracer uptake in the same axial levels (BI–BIV)

Twenty-three months before the patient’s death, cerebral imaging by 2-deoxy-2-[fluorine-18]fluoro-d-glucose integrated with computed tomography-positron emission tomography (^18^F-FDG-PET/CT) was performed according to the following acquisition protocol. Fluorodeoxyglucose (FDG) (±5.3 MBq/kg) was injected intravenously under euglycemic (6-hour fast, capillary blood glucose <140 mg/dl) and standardized resting (eyes open, reduced ambient noise) conditions. After 1 hour of uptake time, images were acquired by positron emission tomography-computed tomography (PET-CT) using a Biograph Duo lutetium oxyorthosilicate PET/CT scanner (Siemens Medical Solutions, Knoxville, TN, USA), followed by dedicated brain PET-CT image acquisition (10-minute acquisition). Next, three-dimensional iterative reconstruction was used for the brain PET images (iterations = 6/subsets = 16/full width at half maximum = 2 mm/matrix = 256). Finally, computed tomographic images were used for attenuation correction of PET data. The cerebral FDG-PET performed 23 months before the patient’s death showed low FDG uptake in the frontal and temporal lobes, especially in the right hemisphere, with relative preservation of the posterior cingulum (Fig. 2a).

**Fig. 3:**
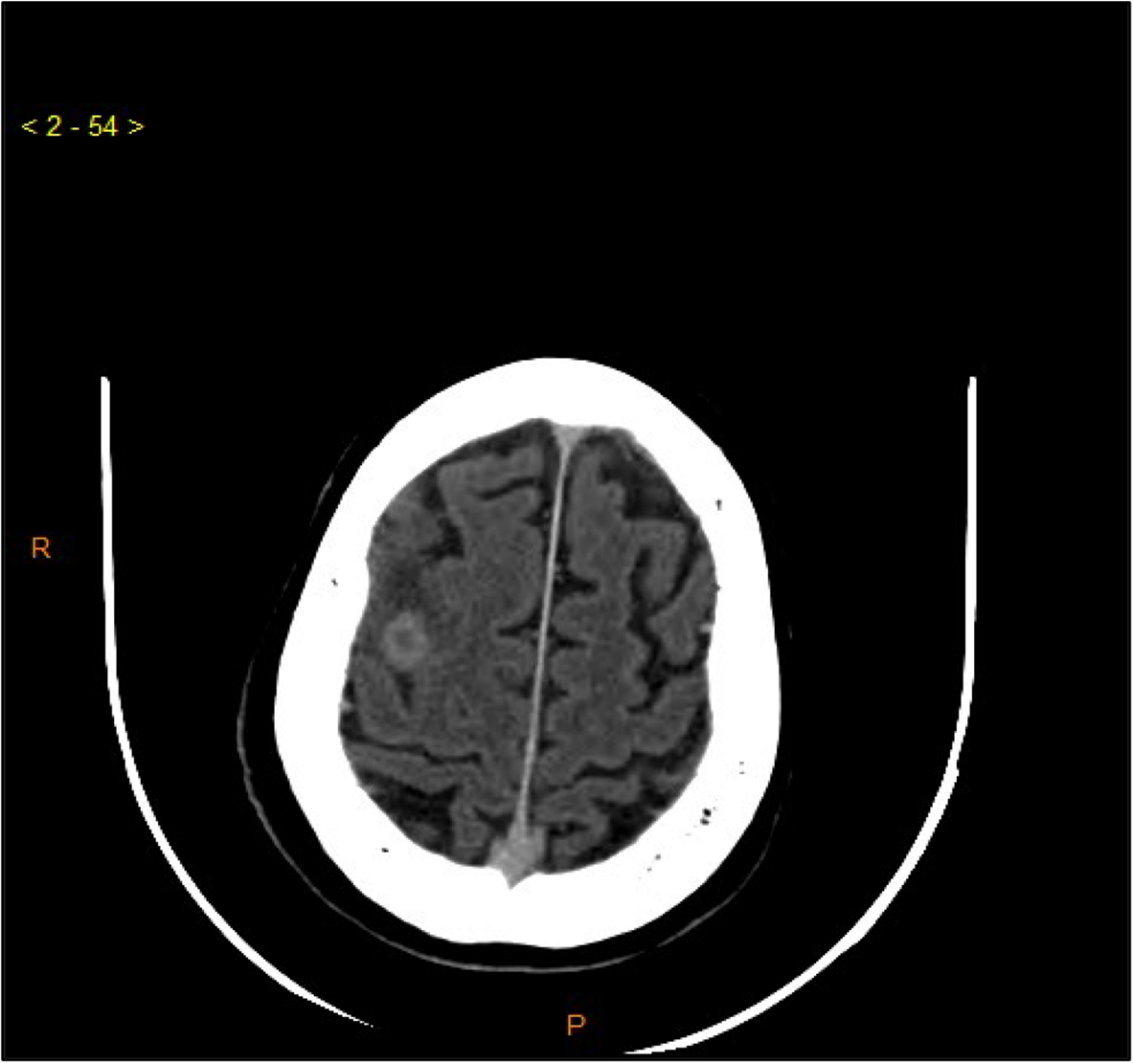
Contrast-enhanced computed tomographic scan of the patient’s brain. Transaxial image shows a brain metastasis in the right hemisphere detected 2 months before the patient’s death

Six months before the patient’s death, he presented with normal-pressure hydrocephalus. A ventricular peritoneum derivation (VPD) was inserted. A small improvement in walking was observed, but it lasted no more than 2 weeks after the VPD implant. Three months before the patient’s death, a second repeat cerebral FDG-PET scan using the same acquisition protocol as before showed diffusely low cerebral glycolytic activity with a marked increment in the severity of the metabolic deficit in the frontal and temporal lobes, as well as hypometabolism involving the parietal lobes and posterior cingulum (Fig. 2b). Three months before the patient’s death, a biopsy done in the right axillary lymph node indicated a metastatic cholangiocarcinoma. The most common type of hilar extrahepatic cholangiocarcinoma is classified into four stages according to the Bismuth classification [5]. In our patient, this classification was considered stage IV. Two months before his death, a contrast-enhanced computed tomographic scan showed brain metastases in the right parietal cortex (Fig. 3).

**Fig. 4:**
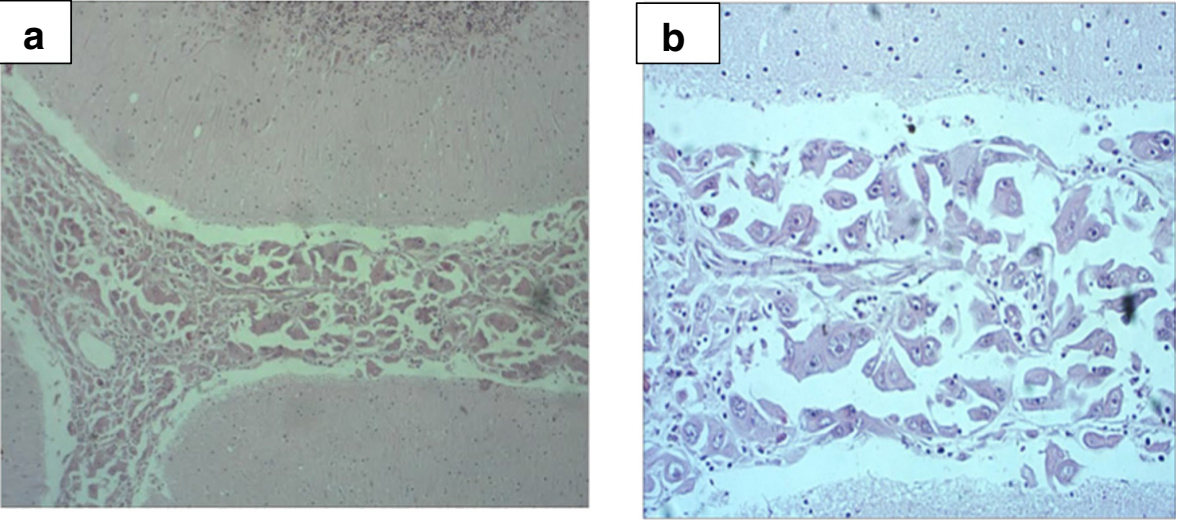
Postmortem analysis. **a** Cerebral metastasis seen at the subarachnoid space (hematoxylin and eosin stain, original magnification ×100). **b** Higher-magnification view the metastatic cells, which show marked pleomorphism, hyperchromasia, and atypia (hematoxylin and eosin stain, ×200)

A postmortem examination showed a cholangiocarcinoma with multiple metastases that included the lungs and lymph nodes. All other organs were studied during the autopsy, and the only primary neoplasia found was the cholangiocarcinoma; the other sites were metastatic, all exhibiting the same morphological aspects as the cholangiocarcinoma. The brain weighed 1300 g, and a catheter was well positioned in the lateral ventricle. There was mild cortical atrophy, *ex vacuo* dilation of the ventricles, and mild focal thickening of the cerebellar leptomeninges, which were infiltrated by the carcinoma, described as neoplastic epithelial cells (Fig. 4). The morphological aspects were similar to the cholangiocarcinoma seen in the liver, histopathologically confirming the brain metastasis. There were no other changes in the cerebral cortex and white matter, except some thickened, hyalinized microvessels in the deep white matter with adjacent gliosis and calcification of vessel walls in the basal ganglia. Axonal peripheral neuropathy associated with microangiopathy, possibly related to diabetes, was also seen.

## Discussion

Our patient showed cognitive symptoms with subacute onset seizures, including frequent autonomic seizures with motor piloerection. Ictal piloerection seizure has been considered the predominant seizure type associated with LE [6]. However, the diagnosis of autoimmune-mediated LE based on the detection of anti-VGKC-Ab is not fully supported by recently published guidelines for diagnosis of autoimmune encephalitis [7]. In fact, the presence of autoantibodies does not always imply an accurate diagnosis. It should be mentioned that recently researchers reported that VKGC antibodies identified by radioimmunoassay in which a complex of brain proteins (KV1.1 and KVI.2) are labeled does not necessarily indicate an absolute specificity for VGKC. The term *voltage-gated potassium channel complex* includes antibodies against LGI1, CASP, and unknown antigens [8]. In contrast to these findings, our patient exhibited a clinical syndrome that allowed the diagnosis of LE to be made. However, it would be of interest to have the results of the complete VGKC complex test. Recently reported information on VGKC indicates that the inclusion of values of VGKC may be of less clinical significance, especially in the context of the absence of LGI1 and CASP antibodies [9]. Therefore, VGKC antibodies may be misnamed, and the diagnosis should rely on the presence of LGI1 and CASP antibodies.

Neuroimaging, especially cerebral FDG-PET, was determinative for the elucidation of our patient’s case. It would be of interest to identify PET cerebral patterns that could lead to an early suspicion of a tumor, such as a cholangiocarcinoma, in patients with rapidly progressive dementia. Because of its size, the tumor may initially remain undetected. The case of our patient may indicate that clinical neurological symptoms, together with specific PET cerebral patterns, may help in the search for small tumors. Considering these findings, sequential whole-body PET scans should be an important tool to be used in vigilance for malignancy at intervals not longer than 1.5 years.

Risk factors frequently associated with cholangiocarcinoma include parasitic diseases involving the biliary tree, preexistent liver and biliary disease, obesity, diabetes, HIV infection, and toxic exposure [4]. Our patient did not have any of these risk factors, except for diabetes. Surgical resection and liver transplant are the effective treatments available [10]. However, only patients with early-stage disease benefit from these treatments. Consequently, an early diagnosis is required. Our patient was in stage IV, which precluded any surgical treatment. Early confirmation of cholangiocarcinomas can be very challenging because these lesions often grow longitudinally along the bile duct rather than in a radial direction away from the bile duct [6]. Imaging techniques such as CT, ultrasound, and magnetic resonance imaging may be of limited sensitivity in these cases [6].

Researchers in most FDG-PET studies of patients with LE have described mesiotemporal hypermetabolism [11]. In contrast, the authors of other reports have described mesiotemporal hypometabolism in patients with LE even several months after symptom onset [11, 12]. The variability in LE imaging data may be due to different autoimmunological mechanisms mediated by a particular autoantibody type [13]. In our patient, hypometabolism was found approximately 2 years before he died.

Different pathological mechanisms underlying LE associated with antibodies against either intracellular or surface antigens have been described after the recent identification of new antineuronal autoantibodies [8]. Previous studies of patients with LE demonstrating hypermetabolism in the mesiotemporal region showed that the patients had positive test results for autoantibodies against intracellular antigens [14, 15]. In contrast, the authors of most reports on patients with LE with autoantibodies against surface antigens have described normal metabolism or hypometabolism in mesiotemporal regions [13]. In this regard, two different pathological mechanisms have been proposed to explain the findings of hypometabolism in the mesiotemporal cortex associated with autoantibodies against surface membrane antigens. First, Hughes *et al*. [16] suggested that a reduction in neuronal activity may be associated with a decrease in the surface density of *N*-methyl-d-aspartate receptors via antibody capping and internalization. Second, Baumgartner *et al*. [13] proposed that PET hypometabolism may be caused by direct blockade of receptors by cell surface antibodies. Furthermore, it is hypothesized that these pathological mechanisms do not induce inflammatory damage, activation of immune cells, or tissue repair that would cause a relevant increase in glucose metabolism.

## Conclusions

The pattern of cerebral hypometabolism, together with clinical findings of LE, should direct the search for a small tumor. Our patient with cholangiocarcinoma also showed very pronounced mesiotemporal hypometabolism, brain atrophy, and clinical symptoms of LE. This case supports continued vigilance in malignancy surveillance of patients with LE. We suggest that any patient presenting with rapidly progressive dementia and cerebral PET abnormalities should be investigated for small tumors, such as a cholangiocarcinoma, using whole-body PET at intervals not longer than 1.5 years.

## Abbreviations

CSF, cerebrospinal fluid; CT, computed tomography; FDG, fluorodeoxyglucose; FDG-PET/CT, 2-deoxy-2-[fluorine-18]fluoro-d-glucose integrated with computed tomography-positron emission tomography; LE, limbic encephalitis; VGKC, voltage-gated potassium channel; VGKC-Ab, voltage-gated potassium channel antibodies; VPD, ventricular peritoneum derivation
